# First records of the cosmopolitan terrestrial slug, *Deroceraslaeve* (O.F. Müller) (Gastropoda, Agriolimacidae) in the Philippines

**DOI:** 10.3897/BDJ.12.e127375

**Published:** 2024-09-05

**Authors:** Veronica B. Tañan, Loel B. Dalan, Sheryll Mae Roy, Augie Fuentes, Irma Tandingan De Ley, Nanette Hope N. Sumaya

**Affiliations:** 1 Department of Biological Sciences, College of Science and Mathematics, Mindanao State University-Iligan Institute of Technology, Andres Bonifacio, Tibanga, 9200 Iligan City, Philippines Department of Biological Sciences, College of Science and Mathematics, Mindanao State University-Iligan Institute of Technology Andres Bonifacio, Tibanga, 9200 Iligan City Philippines; 2 FBL-Nematology Research Group, Center for Biodiversity Studies and Conservation, Premier Research Institute of Science and Mathematics, Mindanao State University-Iligan Institute of Technology, Andres Bonifacio, Tibanga, 9200, Iligan City, Philippines FBL-Nematology Research Group, Center for Biodiversity Studies and Conservation, Premier Research Institute of Science and Mathematics, Mindanao State University-Iligan Institute of Technology Andres Bonifacio, Tibanga, 9200, Iligan City Philippines; 3 Department of Mathematics and Natural Sciences, North Eastern Mindanao State University, Rosario, Tandag City, 8300 Surigao del Sur, Philippines Department of Mathematics and Natural Sciences, North Eastern Mindanao State University Rosario, Tandag City, 8300 Surigao del Sur Philippines; 4 Davao del Sur State College, Digos City, Davao del Sur 8002, Philippines Davao del Sur State College Digos City, Davao del Sur 8002 Philippines; 5 Department of Nematology, University of California-Riverside, Riverside, California, United States of America Department of Nematology, University of California-Riverside Riverside, California United States of America

**Keywords:** *Deroceraslaeve*, mountainous regions, southern Philippines, vegetable pest

## Abstract

The cosmopolitan terrestrial slug, *Deroceraslaeve* (O. F. Müller, 1774), is reported in the Philippines for the first time and characterized through morphology, morphometrics, and cytochrome oxidase subunit I (COI) gene analysis. Slug samples were recovered from two administrative regions in Mindanao, Philippines. In Region X, there were two sites: Misamis Oriental (Gingoog, 664 m a.s.l.; Claveria, 937 m a.s.l.) with farms planted with cabbage (*Brassicaoleracea*), radish (*Raphanussativus)*, and eggplant (*Solanummelongena*); and Bukidnon (Talakag, 1410 m a.s.l.) planted with cabbage. In Region XI, specimens were collected from potted ornamentals in five nurseries along the Kapatagan road, Davao del Sur, 1000-1200 m a.s.l. The external morphology of the specimens matched the published descriptions, and their identity was further confirmed by their partial COI sequences. The obtained COI sequence of the specimen in Region X showed 99-100% similarity with the voucher specimens from Mexico (KX959495, KX959496, KX959497, KX959498, and KX495499); while that of the specimen from Region XI is 100% identical to specimens collected from Japan (MW507142), Canada (MT680918 and MT941436), UK (KF894311), and Vietnam (MT941435 and MT941436). Moreover, *D.laeve* from Region X and Region XI shared 98% similarity with each other. Preliminary surveys show that slug occurrence is prevalent mainly in highland regions of the southern Philippines where specialty crops/high value crops like vegetables and ornamentals are cultivated. Further surveys are essential to confirm any damage that they may cause, their distribution, associated parasites, and pest status in the Philippines.

## Introduction

Slugs are one of the most effective pest groups among terrestrial gastropods (South 2012). The slug morphology evolved from various lineages of land snails that gradually lost their shell through a process known as limacization (Simone 2018). The slug body structure is present among the Stylommatophora (terrestrial snails and slugs) and Systellommatophora (aquatic and terrestrial slugs) clades of the Eupulmonata (Schrödl 2014).

The terrestrial slug *Deroceras* (Gastropoda, Agriolimacidae), is a large genus with approximately 123 species (Wiktor 2000). *Deroceraslaeve* (O.F. Müller, 1774) is one of the most cosmopolitan taxa and has several synonyms of species' names as listed in MolluscaBase. https://www.molluscabase.org/aphia.php?p=taxdetails&id=1002990 (accessed June 19, 2024). This is mostly due to the species' wide geographic spread and variation in body size and coloration (Kennard and Woodward 1926, Wiktor 2000). Whereas the species distribution is believed to be cosmopolitan, its origin remains unclear (Gittenberger et al. 2018). It has long been considered native to the Palaearctic and parts of the Nearctic regions (Wiktor 2000). Furthermore, several previous records suggest a Holarctic origin of *D.laeve* (Sysoev and Schileyko 2009), efficient in colonizing a wide range of habitats due to its vast ecological plasticity. It is also believed that this species was accidentally introduced into other areas before reaching the mountainous regions of the Neotropics in the nineteenth century (Simroth 1910, Anderson 2022). It later spread to the lowlands of South American countries, New Zealand, Australia, and some parts of the Pacific Islands. To date, *D.laeve* is found on all continents except Antarctica (Wiktor 2000, Jordaens et al. 2006).

*Deroceraslaeve*, and a few other members of the genus, are important pests in agriculture and horticulture, causing significant damage through the consumption of plant tissues (Gupta et al. 2023). Various crops are known to be infested with slugs, which includes passion fruit (de Oliveira and Frizzas 2014, Rowson et al. 2014); grapes (Baronio et al. 2014, Rodríguez-Pérez et al. 2019, Das et al. 2020); strawberry (Duthoit 1964, Runham and Hunter 1970, Howlett 2012, Landal et al. 2019, González et al. 2020); vanilla (Vanitha et al. 2011, Velázquez-Montes de Oca et al. 2014); and cabbage, lettuce, carrot, cauliflower, corn, and cucumber (Raut and Panigrahi 1990, Duthoit 1964, Howlett 2012, Hara et al. 2013, Capinera 2020).

Moreover, *D.laeve* is widely known to be a reservoir of diverse nematodes, playing different roles (Gang and Hallem 2016), including those that pose health risks. For instance, they have the potential to spread parasites such as *Angiostrongyluscostaricensis*, which is responsible for abdominal angiostrongyliasis and eosinophilic enteritis in humans when ingested accidentally with vegetable crops (Maurer et al. 2002, Rojas et al. 2021) . Other associated nematode species are *A.vasorum*, affecting the cardiopulmonary system of canids and the respiratory tract of felids (Conboy 2009, Nabais et al. 2014), and *A.cantonensis*, which has been reported to cause eosinophilic meningoencephalitis in Southeast Asia and the Pacific Islands (Wallace and Rosen 1969).

Our communication results from preliminary surveys in the two administrative Regions X and XI in Mindanao island, the Philippines. Additional surveys are underway to determine the presence of other terrestrial slugs in both regions. We herein present the first records of the slug *D.laeve* in the Philippines, along with its essential morphological characteristics and COI gene partial sequences.

## Material and Methods

### Collection areas

Compared to terrestrial snails, slug studies have received significantly less attention as their role in zoonosis or impact on agriculture as pests has yet to be evaluated in the Philippines. The towns of Claveria, Gingoog (in Misamis Oriental), and Talakag (in Bukidnon) are located in Region X, with relatively higher elevations. They are considered fruit baskets in the country (Philippine Statistics Authority (PSA) 2021). Various large and small farms in these areas cultivate crops like cabbage, lettuce, carrot, potato, radish, eggplant, tomato, etc. Based on our interviews, local farmers opt to apply metaldehyde as a molluscicide to control pest populations. Kapatagan, formerly known as Rizal, lies on the eastern part of Mt. Apo, and North of Digos City, Davao del Sur, Region XI. It has a total land area of 8,333.33 hectares comprising 54 puroks/settlement units. Located at a higher elevation and at the foot of Mt. Apo, the country’s highest mountain peak, it was designated an ecotourism site in 2021. Temperature varies little throughout the year: from 23°C to 33°C and is rarely below 22°C or above 36°C; with the wettest season in June, the drier season in December-May, and the least wet days in March. The area within 2-50 miles of Kapatagan is covered by cropland, trees, and water. https://weatherspark.com/y/139108/Average-Weather-in-Kapatagan-Philippines-Year-Round (accessed January 16, 2024). During and following the COVID-19 pandemic in 2020-2023, mountain resorts and ornamental nurseries dotted the road connecting Digos City to Bansalan through Kapatagan and Bansalan-Mt. Apo National Park Road. There has been an increase in human activities including specialty crop/high-value crop farming and establishment of plant nurseries.

### Slug collection

**Figure 1.**
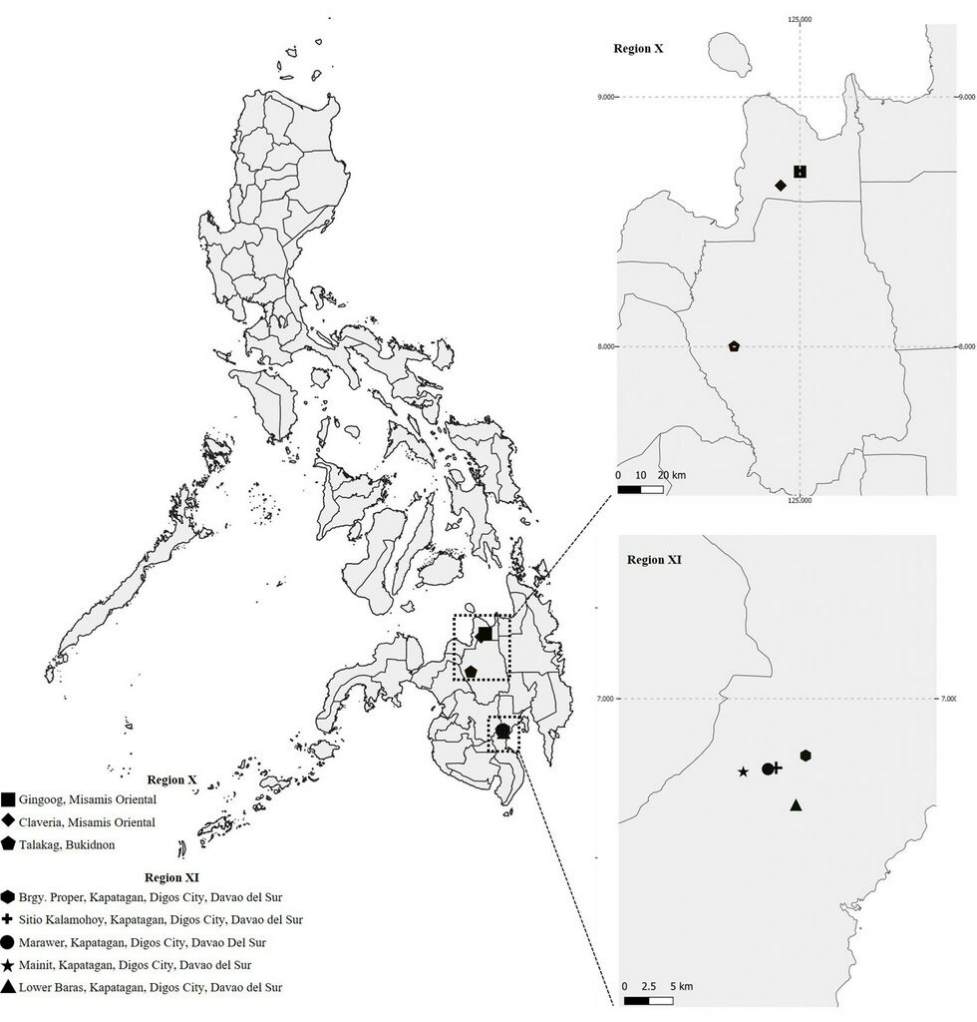
Map of the Philippines showing sites where slugs were found in Region X and Region XI.

**Figure 2.**
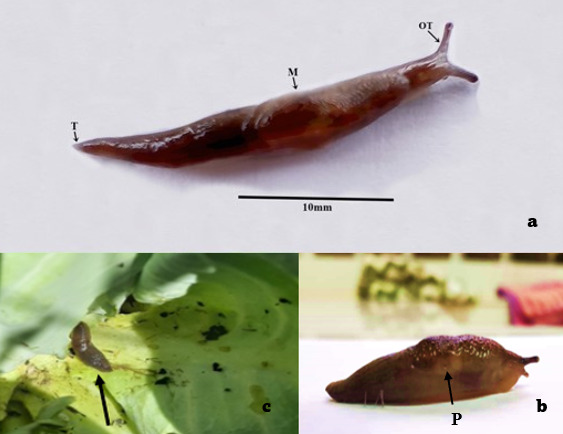
*Deroceraslaeve* from Gingoog (a) showing the ocular tentacle (OT), mantle (M), tail (T), and pneumostome (P); specimen from Claveria, Misamis Oriental (b); and a damaged cabbage leaf (c) from Talakag, Bukidnon, Philippines.

**Figure 3.**
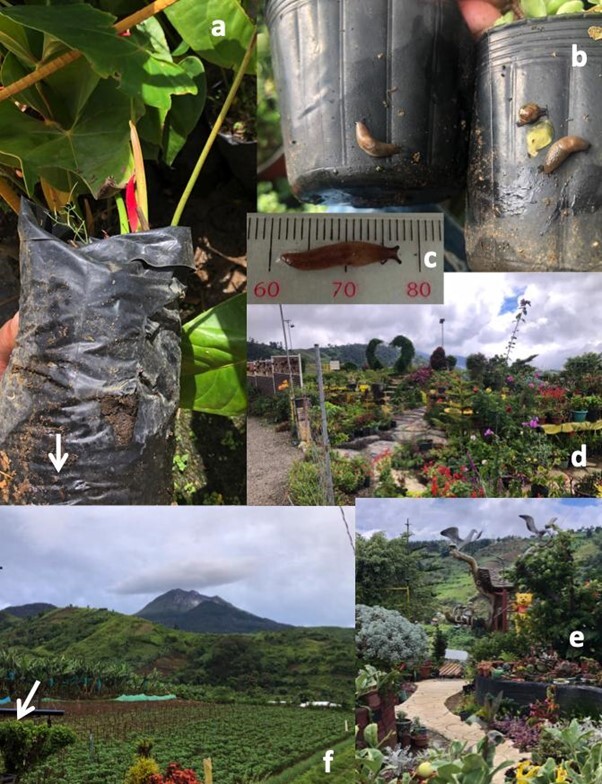
*Deroceraslaeve* (white arrow) outside the plastic pot with damaged *Anthurium* leaves (a); on the sides of potted *Sedum* (b); and a specimen measured on a mm ruler (c). Highly dense, diverse landscaped ornamentals and fruit trees in pots and planted into the ground at Rose Yellow Garden (d and e). Suan Farm with the peak of Mt. Apo in the background; (f) and an arrow indicating the area near the cottage where slugs were recovered. All photographs were taken in Kapatagan, Digos City, Davao del Sur on Nov 7-8, 2023.

Slug specimens in Region X were detected from the three farms of Claveria and Gingoog in Misamis Oriental and Talakag, Bukidnon, Philippines as shown in Fig. 1. More than 40 slug specimens were collected from cabbage (*Brassicaoleracea*), radish (*Raphanussativus)*, and eggplant (*Solanummelongena*) crops during the field sampling in between February to May 2022 (Table 1, Fig. 2). In Region XI, 48 slug specimens were collected from five of six nurseries in Kapatagan, Davao del Sur (Table 1l).There were no *D.laeve* in Cathedral Falls; Tibanga, Iligan City; Lantapan, Bukidnon all of Region X; as well as Eduardo Gamban Nursery in Kapatagan, Digos City, Region XI. At the nurseries, most slugs were collected from under potted succulents arranged on wooden planks and benches on roadside nurseries, plants around two dwellings/cottages as well as on a table under shade trees next to land lying fallow. The succulents were a collection of different types that include *Echeveria*, *Sedum*, *Crassula*, *Pachyphytum*, and *Graptopetalum*; including some potted cacti (Table 1, Fig. 3). Additionally, during these samplings, we found *Laevicaulisalte* Férussac, (1822), *Achatinafulica* Férussac (1821), *Sarasinulaplebeia* Fischer (1868), and *Oxychilusalliarius* (Miller, 1822) in Region X; *L.alte*, *S.pleibea*, *A.fulica*, and *Bradybaenasimilaris* Férussac (1821) in Region XI.

The slugs were carefully handpicked and cleaned to eliminate external debris such as slug feces and tissue fragments of the crops, for further processing and characterization. They were sorted according to species, placed in plastic containers lined with moistened paper towels, provided with carrot discs, and sealed with lids that had tiny perforations.

### Morphological and molecular characterization

All slug specimens were morphologically characterized following the slug identification guide of McDonnell et al. (2009). They were allowed to crawl on a clean tabletop with a ruler, and when slugs stopped moving but extended, they were individually measured and photographed. Key characters such as body color, body texture, total body length, head length, body width, pneumostome or breathing pore, mantle, keel, and tail were distinguished and evaluated for all Gingoog, Claveria, Talakag, and Kapatagan collected specimens. Ten slug specimens were killed by submerging them in a jar filled with cold, sterile water (boiled and cooled down) for 24 hours. They were then transferred to containers with 95% ethanol for voucher storage at the Natural Science Museum of the College of Science and Mathematics (NSM-CSM) and the Flora Biodiversity Laboratory of Premier Research Institute of Technology (FBL-PRISM) with collection number 032022SCC in Mindanao State University - Iligan Institute of Technology.

For molecular analysis, an entire slug specimen each from Gingoog, Misamis Oriental (Region X) and Quinto Farm, Kapatagan, Davao del Sur (Region XI) were placed in separate vials containing 95% ethanol and were sent to the Philippine Genome Center-DNA Core Sequencing Facility (PGC-DCSF) at the University of the Philippines-Diliman, Quezon City. The remainder of the slugs were maintained in carrot discs as previously described until they die for malacoparasitic nematode isolation.

Genomic DNA (gDNA) was extracted following the DNeasy Blood and Tissue Kit (catalog No. 69504) protocol. gDNA purity, integrity, and size were assessed on 1.2% gel with 1kb DNA ladder (Invitrogen) at 100V for 45 minutes. The cytochrome oxidase subunit I gene (COI) was amplified using the universal primer pair LCOI490 (5’-GGTCAACAAATCATAAAGATATTGG-3’) and HCO2198 (5’-TAAACTTCAGGGTGACCAAAAAATCA-3’) (Folmer et al. 1994) with the following cycling parameters: 95 °C 5 min; 30 cycles of 95 °C 1 min, 58 °C 45 sec, 70 °C 1 min, 72 °C 10 min; hold at 4 °C. Capillary bidirectional sequencing was performed at PGC-DCSF. Fluorescent-labeled chain terminator dNTPs with the reaction components viz. amplicons, primers, and ABI BigDye® Terminator v3.1 Cycle Sequencing Kit (Thermo Fisher Scientific) were used. Using a Bio-Rad T100 Thermal Cycler, cycling parameters include pre-hold at 4 °C; 96°C for 1 min; 25 cycles of 96 °C for 10 sec, 50 °C for 5 sec, 62 °C for 4 min and hold at 4 °C. Ethanol precipitation was carried out to remove unincorporated ddNTPs, excess primers, and primer dimers. Capillary electrophoresis was performed on the ABI 3730xl DNA Analyzer using a 50 cm 96-capillary array, POP7TM Polymer, and 3730xl Data Collection Software v3.1. Base calling was done using the Sequencing Analysis Software v5.4.

### Sequence alignment and phylogenetic analysis

The obtained COI sequences were trimmed and assembled using BioEdit v7.2.5 (Hall 1999) and CodonCode Aligner 7.0.1 (CodonCode Corporation, 101 Victoria Street, Centerville, MA 02632, USA) then BLASTn-searched at the National Centre for Biotechnology (NCBI). The sequences were compared with the nearest relative sequences in the database based on percentage similarities. Additional sequences of related taxa were downloaded from Genbank, and were aligned and trimmed using MUSCLE in MEGA v11 (Tamura et al. 2021). The phylogenetic tree was generated in MrBayes v3.2.7 (Ronquist et al. 2012) using the best fit HKY + G model as subjected to jModeltest 2.1.10 analysis (Darriba et al. 2012). Analyses were run for 1 × 10^6^ generations (4 runs) with Monte Carlo Markov chains sampled for every 100 generations, wherein 20% of the converged runs were considered burnins (Huelsenbeck and Ronquist 2001). The number of base differences per sequence from between sequences was calculated with pairwise distance analysis in MEGA v11 (Tamura et al. 2004).

## Results

### Morphological and morphometrical characterization

Eighty-eight slug specimens were found in Regions X (40) and XI (48). *Deroceraslaeve* were seen feeding on leaves (reducing the leaf surface area) and leaving holes along with feces on various crops or hiding under, or crawling on, the side of the pots. For Region X, only 33 specimens were further examined and measured. A total of 18 specimens of *D.laeve* were examined from cabbage, ten from radish, and ten from eggplant farms in Gingoog and Claveria, Misamis Oriental, while five samples of *D.laeve* were examined from a cabbage farm in Talakag, Bukidnon. In Region XI, a slug (Nora Gabito roadside nursery) went missing by the time we reached the laboratory so only 47 were measured. Slugs showed slight differences in external appearance and sizes, however, the key characters typical of the species were observed.

*Deroceraslaeve* is small and slim, up to ~25 mm in length and ~6 mm in width (Table 2 and Table 3), and secretes clear mucus. The body is cylindrical and somewhat broader in the middle. The mantle is wrinkled and measures less than half its body length that ranges between 9-10mm. The head region, which comprises a pair of ocular and sensory tentacles, averaged 3 mm. The tail is smooth and measures 10-11 mm from the posterior of the mantle to the tail tip. Body length of slugs from Kapatagan, Davao del Sur were shorter than those observed in Region X, and there were variations also among the nurseries, ranging from 14-24 mm long and 3.0-6.12 mm wide. However, these differences in measurements are likely attributed to differences in life stages, the smaller ones being juveniles; among other factors.

### Molecular characterization and phylogenetic analysis

The generated COI sequences from the slug samples from Gingoog, Misamis Oriental (GMO) and Kapatagan, Davao del Sur (KDS) were submitted to NCBI (accession OP836297 and PP152234). *Deroceraslaeve* GMO (OP836297) was 100% identical with *D.laeve* KX959498 and 99% similar with *D.laeve* KX959497, KX959499, KX959496, and KX050495 (1-2 bp difference) from Mexico (Araiza-Gómez et al. 2017). Moreover, *D.laeve* GMO (OP836297) shared 98% similarity with *D.laeve* (MT941435 and MT941436) from Vietnam (Dedov et al. 2020). On the other hand, *D.laeve* KDS (PP152234) had 100% similarity with *D.laeve* MW507142 from Japan (Matsushima and Haga 2021), *D.laeve* MT680918 and MG422202 from Canada (Brophy et al., unpublished; Deeward unpublished), and *D.laeve* KF894311 from UK (Rowson et al. 2014). Furthermore, *D.laeve* KDS (PP152234) had higher similarity of 99% (1 bp difference) with the other Southeast Asian *D.laeve* MT94135 and MT94136 (Dedov et al. 2020). Between the two populations GMO and KDS (OP836297 and PP152234) in this study, they shared 98% similarity with 4 bp difference.

**Figure 4.**
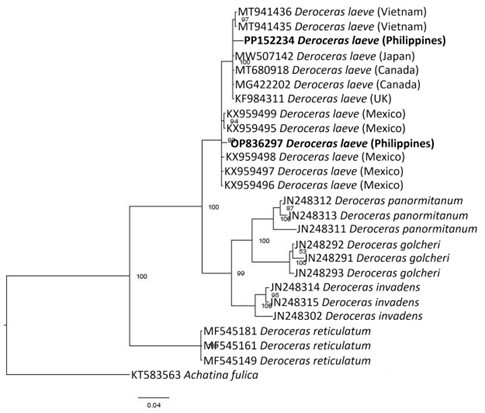
Phylogenetic position of *Deroceraslaeve* (OP836297) from Gingoog, Misamis Oriental and *D.laeve* (PP152234) from Kapatagan, Davao del Sur, Philippines as inferred from COI gene sequences. The scale bar represents the estimated number of base substitutions per site among sequences. Node values indicate the % posterior probability values.

Bayesian analysis of GMO and KDS with other related member taxa shows a distinct, well-supported clade with *D.reticulatum* as the basal taxon (percent probability value 100%). The two Philippine specimens formed a sister clade alongside their closest relatives (Fig. 4). This was supported by pairwise distance analysis using the number of base differences between sequences. GMO OP836297 is closest to Mexican populations KX959495-KX959499, with an average base difference of 0.80 between sequences; whereas KDS PP152234 is most closely related with *D.laeve* from Japan (MW507142), population from Canada (MT680918 and MG422202), from UK (KF894311), and Vietnam (MT94136 and MT94135) with no base difference between sequences. The two Philippine populations GMO and KDS had the highest number of base differences (3 bases) between them (Table 4).

## Discussion

Slugs are among the most abundant invertebrates in northwestern Europe, typically observed in gardens and around buildings. There are 36 species of slugs in Britain and Ireland, which includes the genus *Deroceras* of the Agriolimacidae family (Anderson 2005, Rowson et al. 2014). *Deroceraslaeve* is native throughout Europe and the Arctic (Rowson et al. 2014), as well as Asia and North America and have been brought to other parts of the world (Wiktor 2000, Grimm et al. 2010). This slug has been recorded in gardens, farms, forests, and metropolitan areas in Asian countries such as Bhutan, China, India, Israel, Pakistan, Nepal, India, Sri Lanka, Taiwan, Japan, Vietnam, and Malaysian Borneo (Wiktor 2000, Naggs et al. 2003, Wiktor 2003, Hlaváč 2004, Surya Rao et al. 2004, Bössneck 2006, Tsai and Wu 2008, Jayashankar et al. 2012, Budha et al. 2015, Seebens et al. 2017, Gittenberger et al. 2018, Dedov et al. 2020, Liew and Foon 2022). Moreover, *D.laeve* serves as an intermediate host for *Angiostrongyluscantonensis*, a metastrongylid worm that causes disease in humans (Maurer et al. 2002).

*D.laeve* has been observed worldwide and variation in its size are due to differences in developmental stages (Shirley et al. 2020) among others. *D.laeve* across the whole Europe has a body size of 15 to 25 mm (Rowson et al. 2014). *D.laeve* from Vietnam, Bhutan, and Malaysia are smaller at 22 mm long (Schilthuizen and Liew 2008, Gittenberger et al. 2018, Dedov et al. 2020) compared to the samples from North America that are more than 25 mm long making it sometimes comparable to *D.invadens* (Hutchinson et al. 2014). The *D.laeve* recently recorded from the southern Philippines has a 23 mm mean body length for Region X, and smaller (15-24 mm to exclude juvenile outliers) for Region XI, which is closely similar in size to the population from Vietnam (Dedov et al. 2020). In ethanol-preserved specimens, the total body length of those from Vietnam ranged from 16-18mm, 18-22mm from Mexico, and 19-24 mm from the live specimens in the Philippines. Overall, other character sizes are within the range with similar morphological descriptions such as grey to dark-brown live pigmentation, black ocular tentacles, wrinkled mantle, smooth tails, and keel. As mentioned earlier, such differences can be attributed mainly to differences in life stages. However, even if adults are measured, differences are expected and they can be attributed to several factors, such as geographical locations, adaptation strategies, feeding preferences, changes in habitat temperature, and many more (Berman et al. 2011).

The collected specimens had gigantic mantles and showed delicate wrinkles visible in front of live specimens, similar to the species from other countries. The back slopes evenly towards the tail or is squared off and moderately truncated. A small keel closes to the tail end (Rowson et al. 2014). It is variable in ground color, ranging from an opaque chestnut to grayish brown to chocolate-brown to gray. On close examination, the back is sparsely mottled with a darker shade, which is not easily visible to the naked eye. Confirmation of the species identity is supported by their partial COI sequences. Both sequences are the first record obtained for *D.laeve* from the Philippines which showed degrees of similarities from the available sequences in NCBI GenBank.

There were a number of base differences in the COI sequences between the two Philippine *D.laeve* specimens. The variations could also help explain why the two populations are not in the same clade, instead forming a sister clade with each other. *Deroceraslaeve* KDS (PP152234) is closely related to the population recorded from Japan, Canada, UK, and Vietnam (Rowson et al. 2014, Dedov et al. 2020, Matsushima and Haga 2021, Deeward unpublished) whilst *D.laeve* GOM (OP836297) is closely related to population from Mexico (Araiza-Gómez et al. 2017). This further supports the wide range distribution of *D.laeve* worldwide as invasive species.

Based on our preliminary surveys, slugs are most commonly found in the highlands of the southern Philippines, where specialty/high value crops like vegetables and ornamentals are cultivated. More surveys are needed to confirm the extent of any damage they may cause, their distribution, pest status, and associated parasites in the Philippines.

## Conclusions

Specimens of the invasive *Deroceraslaeve* were recovered from infested field cabbage, radish, and eggplant plantations as well as potted succulents and ornamentals. The external morphological characters of the specimens concur with published descriptions and species identity is supported by their COI mitochondrial DNA sequence. This agriolimacid slug has been documented worldwide establishing themselves as agricultural pests. Our surveys constitute the beginning of additional surveys to determine the occurrence of terrestrial gastropods and assess their potential pest status in the Philippines. This data represents the first record of *D.laeve* in the Philippines, extending its geographical distribution and habitat range in Asia.

## Availability of materials/data

The generated partial COI mDNA sequences are deposited in NCBI.

## Figures and Tables

**Table 1.** *Deroceraslaeve* collected from, geographical locations, and associated plants in Regions X and XI, Mindanao island, the Philippines.

	**Address/Location**	**GPS**	**Elevation (meters above mean sea level)**	**Presence of *D.laeve***	**Associated Plants**
**Region X**					
Cathedral Falls	Kapatagan, Lanao del Norte	7°52'13"N 123°46'31"E	290	-	*Cucurbitamaxima*
Hidden Mickey Resort,	Gingoog, Misamis Oriental	8°42'02.5N 125°00'00.8"E	664	+	*Brassicaoleracea*
Barangay Luna	Claveria, Misamis Oriental	8°38'43"N 124°55'51"E	937	+	*Raphanussativus*, *Solanummelongena*
Tibanga	Iligan City	8°14'23"N 124°15'9"E	860	-	*Ipomoeabatatas*
Talakag	Bukidnon	8°00'26.3"N 124°44'56.8"E	1410	+	*Brassicaoleracea*
Lantapan	Bukidnon	8°2'16"N 124°54'8"E	1200	-	*Brassicarapa*, *Daucuscarota*
**Region XI**					
Nora Gabito flower nursery	Lower Baras, Kapatagan, Digos City, Davao del Sur	6°54'04.9"N 125°20'08.1"E	1000	+	Assorted succulents *Echeveria*, *Sedum*, *Crassula*
Eduardo Gamban nursery	Purok 2, Kinambulan, Kapatagan, Digos City Davao del Su	6°93'61.1"N 125°29'05.6"E	1000	-	Cymbidium orchids, assorted succulents *Impatiens*, *Anthurium*, Philodendron, *Pothus*
Quinto’s farm	Kalamohoy, Kapatagan, Digos City, Davao del Sur	6°56'08.9"N 125°19'00.4"E	1100-1200	+	Assorted succulents *Echeveria*, *Sedum*, *Crassula*, *Pachyphytum*, and *Graptopetalum*
Gil Suan cottage farm	Marawer, Kapatagan, Digos City, Davao del Sur	6°56'04.3"N 125°18'34.2"E	1000	+	Potted *Echeveria*
Renante Cadona succulent collection	Barangay proper, Kapatagan, Digos City Davao del Sur	6°56'49.3"N 125°20'40.6"E	1000	+	Potted *Echeveria*, *Sedum*
Rose Yellow Garden	Mainit, Kapatagan, Digos City, Davao del Sur	6°55'54.5"N 125°17'11.4"E	1300	+	Assorted succulents *Echeveria*, *Sedum*, *Crassula*, *Pachyphytum*, *Graptopetalum* and various rare imported ornamental plants

**Table 2.** Measurements of the key morphological characters of *Deroceraslaeve* collected from the different crops of Region X, Philippines. Values are in millimeters (mm), which represents the mean ± standard deviation and range of values.

***D.laeve* characteristics**	**Gingoog and Claveria, Misamis Oriental**			**Talakag, Bukidnon**
	*Brassicaoleracea* n = 18	*Raphanussativus* n = 10	*Solanummelongena* n = 10	*Brassicaoleracea* n=5
**Body length (mm)**	23.82 ± 3.40 (18.5-31)	23.27 ± 3.18 (18-28)	23.19 ± 2.04 (19-25.2)	23.64 ± 1.62 (21.3-25.6)
**Width (mm)**	6.22 ± 0.80 (5-7)	6.27 ± 0.76 (5-7.2)	6.42 ± 0.73 (5.5-7.4)	6.12 ± 0.83 (4.9-6.9)
**Head (mm)**	2.99 ± 0.58 (1.9-3.8)	2.45 ± 0.40 (1.8-3.3)	2.25 ± 0.36 (1.6-2.8)	2.84 ± 0.23 (2.5-3.0)
**Mantle (mm)**	9.96 ± 1.65 (7.4-13.5)	9.82 ± 1.60 (7-12.2)	9.73 ± 1.02 (7.4-11.2)	9.5 ± 0.60 (8.7-10.0)
**Tail (mm)**	10.9 ± 1.41 (8.6-14.2)	11 ± 1.35 (8.5-12.4)	11.21 ± 0.87 (10-12.6)	10.78 ± 1.28 (9-12)

**Table 3.** Measurements of the key morphological characters of *Deroceraslaeve* collected from the different ornamental nurseries in Kapatagan, Region XI, Philippines. Values are in millimeters (mm), which represents the mean ± standard deviation and range of values.

***D.laeve* characters**	**Nora Gabito flower nursery** n=2	**Quinto’s farm** n=31	**Gil Suan farm** n=2	**Renante Cadona succulents** n=2	**Rose Yellow garden** n=11
**Body length (mm)**	14.0	16.7 ± 4.3 (8.0-25.0)	24.0 ± 1.4 (23.0, 25.0)	21.5 ± 3.5 (19.0, 24.0)	15.0± 5.7 (6.0-26.0)
**Width (mm)**	3.5	3.4 ± 0.8 (2.0-5.0)	4.5 ± 0.7 (4.0, 5.0)	3.0 ± 0.0 (3.0, 3.0)	6.12 ± 0.83 (2.0-4.0)
**Head (mm)**	1.5	2.6 ± 0.7 (1.0-4.0)	3.0 ± 0.0 (3.0, 3.0)	2.5 ± 0.7 (2.0, 3.0)	2.1 ± 0.5 (1.0-2.5)
**Mantle (mm)**	6.0	6.7 ± 1.8 (3.0-10.0)	9.5 ± 0.0 (9.5, 9.5)	13.0 ± 2.8 (11.0-15.0)	5.8 ± 2.5 (2.0-10.0)
**Tail (mm)**	6.5	7.4 ± 2.4 (2.5-11.0)	11.5 ± 1.4 (10.5, 12.5)	3.0 ± 1.4 (2.0, 4.0)	7.1 ± 2.9 2.5-13.5)

**Table 4.** Pairwise distance analysis showing the number of base difference per sequences from between sequences between *Deroceraslaeve* (OP836297 and PP152234) from Gingoog, Misamis Oriental and Kapatagan, Davao del Sur, Philippines with other *D.laeve* specimens.

		1	2	3	4	5	6	7	8	9	10	11	12	13
1	PP152234*Deroceraslaeve* KDS (Philippines)													
2	OP836297*Deroceraslaeve* GMO (Philippines)	3.00												
3	MG422202*Deroceraslaeve* (Canada)	0.00	3.00											
4	MT680918Deroceraslaeve (Canada)	0.00	3.00	0.00										
5	KF894311*Deroceraslaeve* (UK)	0.00	3.00	0.00	0.00									
6	MW507142*Deroceraslaeve* (Japan)	0.00	3.00	0.00	0.00	0.00								
7	MT941436*Deroceraslaeve* (Vietnam)	0.00	3.00	0.00	0.00	0.00	0.00							
8	MT941435*Deroceraslaeve* (Vietnam)	0.00	3.00	0.00	0.00	0.00	0.00	0.00						
9	KX959498*Deroceraslaeve* (Mexico)	3.00	0.00	3.00	3.00	3.00	3.00	3.00	3.00					
10	KX959497*Deroceraslaeve* (Mexico)	2.00	1.00	2.00	2.00	2.00	2.00	2.00	2.00	1.00				
11	KX959499*Deroceraslaeve* (Mexico)	2.00	1.00	2.00	2.00	2.00	2.00	2.00	2.00	1.00	0.00			
12	KX959496*Deroceraslaeve* (Mexico)	2.00	1.00	2.00	2.00	2.00	2.00	2.00	2.00	1.00	0.00	0.00		
13	KX959495*Deroceraslaeve* (Mexico)	2.00	1.00	2.00	2.00	2.00	2.00	2.00	2.00	1.00	0.00	0.00	0.00	
